# Update of the human and mouse *SERPIN* gene superfamily

**DOI:** 10.1186/1479-7364-7-22

**Published:** 2013-10-30

**Authors:** Claire Heit, Brian C Jackson, Monica McAndrews, Mathew W Wright, David C Thompson, Gary A Silverman, Daniel W Nebert, Vasilis Vasiliou

**Affiliations:** 1 Department of Pharmaceutical Sciences, Molecular Toxicology and Environmental Health Sciences Program, University of Colorado Anschutz Medical Center, Aurora, CO 80045, USA; 2 Mouse Genome Informatics, The Jackson Laboratory, 600 Main Street, Bar Harbor, ME 04609, USA; 3 HUGO Gene Nomenclature Committee (HGNC), European Bioinformatics Institute, EMBL-EBI, Wellcome Trust Genome Campus, Cambridge CB10 1SD, UK; 4 Department of Clinical Pharmacy, University of Colorado Anschutz Medical Center, Aurora, CO 80045, USA; 5 Department of Pediatrics and Cell Biology, University of Pittsburgh School of Medicine, Children's Hospital of UPMC, Pittsburgh, PA 15212, USA; 6 Department of Environmental Health and Center for Environmental Genetics, University of Cincinnati Medical Center, Cincinnati, OH 45267-0056, USA

**Keywords:** Serpins, Serine protease inhibitor, Chaperone, Blood clotting, Thrombolysis, Complement, Cell death, Metastatic cancer

## Abstract

The serpin family comprises a structurally similar, yet functionally diverse, set of proteins. Named originally for their function as serine proteinase inhibitors, many of its members are not inhibitors but rather chaperones, involved in storage, transport, and other roles. Serpins are found in genomes of all kingdoms, with 36 human protein-coding genes and five pseudogenes. The mouse has 60 *Serpin* functional genes, many of which are orthologous to human *SERPIN* genes and some of which have expanded into multiple paralogous genes. Serpins are found in tissues throughout the body; whereas most are extracellular, there is a class of intracellular serpins. Serpins appear to have roles in inflammation, immune function, tumorigenesis, blood clotting, dementia, and cancer metastasis. Further characterization of these proteins will likely reveal potential biomarkers and therapeutic targets for disease.

## Introduction

Serpins represent the largest and most functionally diverse family of protease inhibitors. The name serpin originates from the first described function of this family, *viz.*, **ser**ine **p**roteinase **in**hibitors. In their native state, serpins exist as monomeric proteins. Most serpin family members inhibit serine proteinases of the chymotrypsin family [1], thereby inhibiting proteolytic cascades. However, some serpins exhibit functions unrelated to inhibition of catalytic activity, such as hormone transport and other mechanisms.

Approximately 1,500 serpin sequences have been identified; they are found in the genomes of all five kingdoms [2]. There are 36 identified human putatively functional protein-coding genes [3]. The serpin superfamily is divided into groups called clades according to their sequence similarity. Clades are classified as A–P, with clades A–I representing human serpins [4].

**Figure 1:**
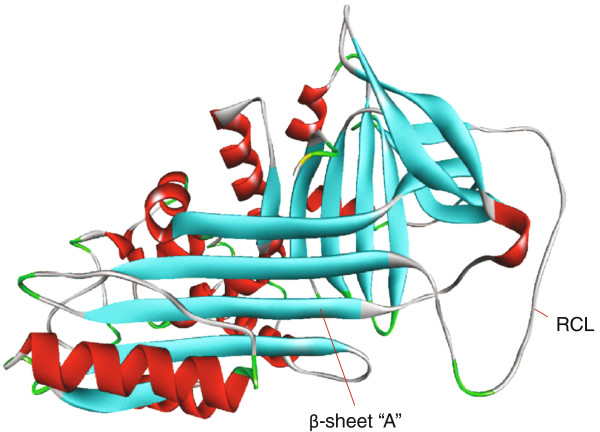
**Native SERPINA1.** Native SERPINA1 with labeled structural elements: β sheet a and reactive center loop (RCL); α helices in red, β sheets in turquoise, turns in green. (Adapted from PDB 1HP7).

Serpins have well-conserved secondary structures with an exposed reactive center loop (RCL) (Figure 1), which interacts with the protease active site to inhibit protease activity [5]. The ability for serpins to undergo conformational change is crucial for their function, in which serpins act via a suicide substrate inhibitory mechanism [2,4]. Although most serpins selectively inhibit serine proteases, some inhibit cysteine proteases, such as caspases and cathespins; others perform hormone transport and blood pressure regulation [4]. Serpins play important physiological roles in hormone transport, corticosteroid binding, coagulation, and blood pressure regulation.

### Serpin nomenclature

**Figure 2:**
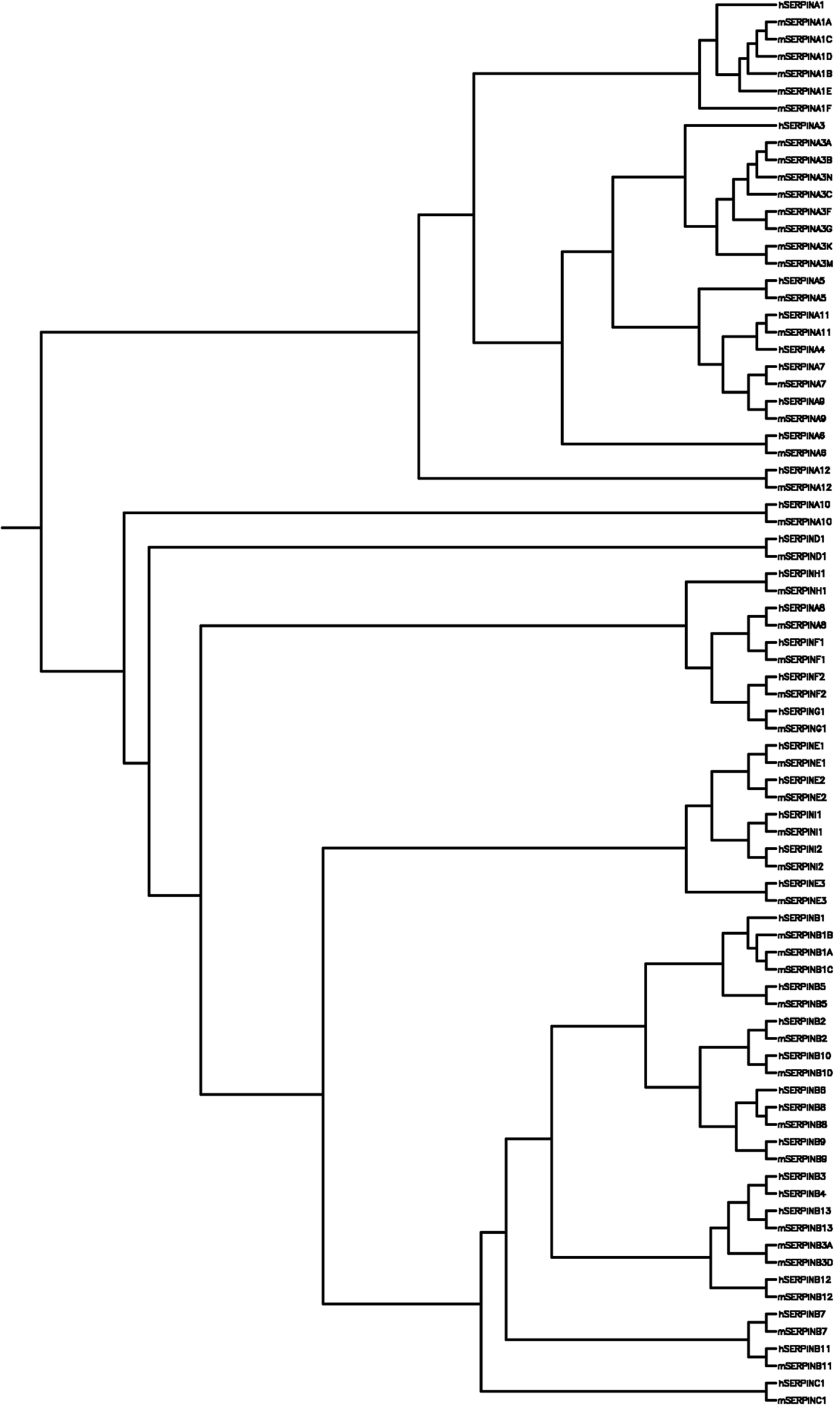
**SERPIN phylogenetic tree.** Phylogenetic tree of human and mouse serpin proteins. Protein sequences were aligned using TCOFFEE and analysed using neighbour-joining methods with 10,000 bootstrap replicates in the Phylip package.

Initially named for tissue location or function (Table 1), a nomenclature committee convened in 1999 with the goal of standardizing serpin gene nomenclature [4]. ‘*SERPIN*’ was designated as the gene symbol for humans and other species because it is well known and used in the literature and as a keyword [4]. Serpins were not named for activity or function due to the diversity of member structure and tissue distribution. In 2005, proteinase in human gene names was replaced with the term peptidase; however, ‘serpin’ remains the stem because the name was designated prior to this change. The current classification of serpins involves division into clades that are based on phylogenetic relationships (Figure 2). There are 16 clades labeled A–P. Human serpins are represented in the first nine clades (i.e., A–I), with a variety of members being in each clade. Clades are phylogenetically unique and it is important to recognize that no relationships between the clade letters are implied by their order [4]. Some serpins are classified as orphans because they do not group with any other clade. It is likely that they will form clades as new serpins are identified. An example to help illustrate the nomenclature would be α-1-antitrypsin. This was assigned to the first clade, giving it the symbol *SERPINA1* with the ‘A’ referencing the clade and the ‘1’ referencing the gene number within the clade [4].

**Table 1:** SERPIN aliases and function

**Clade name**	**Clade**	**Serpin gene name**	**Known aliases**	**Biological function**	**References**
Alpha 1 proteinase inhibitor antitrypsin	A	*SERPINA1*	Alpha 1PI	Inflammation, complement activation, apoptosis	17
		*SERPINA2*			
		*SERPINA3*	Alpha1 ACT	Apoptosis, Alzheimer’s disease, prohormone conversion, inflammation and complement activation	3,16
		*SERPINA4*	PI4, KST, KAL	Kidney function, inflammation, and complement activation	18
		*SERPINA5*	PCI	Coagulation, inflammation, complement activation, sperm development	
		*SERPINA6*	CBG	Hormone transport	16
		*SERPINA7*	TBG	Hormone transport	
		*AGT*	SERPINA8	Blood pressure regulation, renal development	19
		*SERPINA9*	Centurin	B cell development	3
		*SERPINA10*	PZI	Inhibition of activated factors Z and XI	3
		*SERPINA11*			
		*SERPINA12*	Vaspin	Inhibits kallikrein; unknown role in insulin sensitivity	20,21
		*SERPINA13P*			
		*SERPINB1*	PI2, LEI, MNEI, EI	Inflammation, complement activation	3
		*SERPINB2*	PAI2, placental PAI, monocyte ARG serpin, PLANH2	Fibrinolysis, elastase inhibitor	3
ov Serpins	B	*SERPINB3*	SCCA1, SCC	Inhibition of cathepsins, tumor promotion	32
		*SERPINB4*	SCCA2, PI11	Inhibition of cathepsins and chymase	33
		*SERPINB5*	Maspin, PI5	Tumor cell invasion, angiogenesis	3
		*SERPINB6*	DFNB91, PI6, CAP	Cathepsin inhibitor	34
		*SERPINB7*	Megsin	Renal development, mesangial cell proliferation	35
		*SERPINB8*	PI8, CAP2	Uncharacterized	3
		*SERPINB8P1*			
		*SERPINB9*	PI9, CAP3		
		*SERPINB10*	PI10, BOMAPIN	Hematapoetic and myeloid development	35
		*SERPINB11*	EPIPIN	Uncharacterized	36
		*SERPINB12*	YUKOPIN	Trypsin inhibition	37
		*SERPINB13*	PI13, headpin, HUR7, hurpin		
Antithrombin	C	*SERPINC1*	AT3, ATIII, antithrombin3	Coagulation, angiogenesis	38
Heparin cofactor	D	*SERPIND1*	HCFII, HCF2, heparin cofactor II, HLS2	Coagulation	40
Nexin/plasminogen activator inhibitor 1	E	*SERPINE1*	PAI, PLANH1	Angiogenesis, fibrinolysis	3
		*SERPINE2*	PI7, GDN, ‘glial-derived nexin 1,’ nexin, PN1, PNI	Neurotrophic factor	41
		*SERPINE3*			
Alpha 2 antiplasmin pigment epithelium derived factor	F	*SERPINF1*	PEDF	Neurotrophic factor, angiogenesis	16
		*SERPINF2*	Alpha 2AP, PLI, A2AP, AAP, ‘alpha-2-antiplasmin,’ ALPHA‒2‒PI, ‘alpha‒2‒plasmin inhibitor,’ API	Fibrinolysis	3
C1 inhibitor	G	*SERPING1*	C1NH,C1–INH, C1IN, HAE1, HAE2, ‘plasma protease C1 inhibitor’	Microbial infection	42
Heat shock protein	H	*SERPINH1*	CBP1, CBP2, collagen, HSP47	Chaperone	43
Neuroserpin	I	*SERPINI1*	Neuroserpin, PI12	Neurotrophic factor	46
		*SERPINI2*	Pancpin, PI14, TSA2004, MEPI	Tumor cell invasion	47

SERPINS are divided into clades and gene name with known aliases and biological function are provided. Alternative name information was determined using HGNC (http://www.genenames.org) and/or MGI (http://www.informatics.jax.org).

### Structure function

Serpins have a metastable structure that is required for their function. It consists of a highly conserved secondary structure with three β-sheets (A, B, and C), nine α-helices and a RCL (Figure 1), which serve as bait for target proteases [4,6]. Well-conserved throughout the serpin family, the tertiary structure of scaffold allows for a conformational change critical to protease inhibitor activity [4]. In their native state, serpins exist as monomeric proteins. A serpin molecule consists of a single 330- to 500-amino acid polypeptide chain that has conserved secondary helices and sheets. To inhibit proteolytic activity, the serpin acts as a suicide substrate for the protease [4]. This is accomplished by the RCL of the serpin interacting with the protease's active site [6].

Serpins can exist in several forms, *viz.*, active, latent, cleaved, delta, and polymeric. Each form is defined by the RCL, which is the moiety required for inhibitory activity. The active form (or the native state) has an exposed RCL that allows it to interact with the protease. The RCL forms an exposed extension located above the molecule. Following proteolysis, the amino acid terminus of the RCL inserts into the A β sheet forming a fourth strand. This process is called the ‘stressed (S) to relaxed (R) transition’ [3] used to inhibit proteases, resulting in the cleaved form. The cleaved form is necessary for inhibition of proteases resulting in an irreversible covalent complex with the target protease thus inactivating both the serpin and the target. Some serpins bind cofactors and/or glycosaminoglycans to maximize protease inhibition, which can vastly increase inhibitory potential [7].

The native form of serpins has low thermal stability indicating that it is not the most stable conformation; rather, native serpins are metastable. However, not all serpins undergo this transition. Serpins can transition to the latent form from the active form and back to the active form from the latent form. The latent form does not possess inhibitory activity but it can convert to the active form through denaturation and refolding [4]. Consequently, it can be considered a control mechanism in regulating homeostasis for certain serpins [3]. Alternatively, the latent state caused by a mutation can be pathological [3].

The delta form is an intermediate conformation between latent and native state where the RCL inserts into the A β sheet and one of the helices unwinds and completes hydrogen bonding of the β sheet [3]. Little is known about the function of this conformation; however, it is likely that this favors polymeric or latent conformation transition rather than native. The polymeric form has a loop sheet mechanism whereby the RCL that would be inserted into the same serpin is instead inserted into the A β sheet of another serpin forming a long chain of these molecules [3]. However, this mechanism of polymerization has recently been challenged in favor of that of a domain-swapping model [8]. Serpins are unique in that their native state (active form) is not the most kinetically stable; rather, it is ‘metastable’. By incorporating the RCL into their A β sheet, either by cleavage for inhibition of target protease or spontaneous latency, they become more stable [9]. For an excellent minireview on kinetics of serpins, see Silverman et al. [4].

### Evolution

Whereas serpins have highly conserved secondary and tertiary structures upon which they are grouped, they often share little amino acid sequence similarity. They do, however, share a highly conserved core, especially in the shutter domain including Ser56 and Ser53 [10], which is thought to be critical in determining tertiary structure and conformational flexibility.

Due to the numerous, yet distinct, processes regulated by serpins and their widespread functions, serpins offer a unique perspective for protein evolution. Members of the serpin family tend to group phylogenetically by species rather than by function. Therefore, evolution of the serpin family was likely driven by speciation to fill their physiological roles rather than by coevolution with the serine proteases (which group by function) [10]. Numerous serpin genes are also found in clusters on the same chromosomes, reflecting earlier gene-duplication events and potentially indicating a common precursor [11,12]. Interestingly, these genes are functionally divergent, despite their chromosomal proximity [7]. In addition, serpins have distinct patterns of introns and exons. These patterns may contain information regarding phylogenetic signals and be evolutionarily related based on relative intron positioning [13,14].

The distribution of serpins in eukaryotes suggests that they arose early in eukaryotic evolution [1]. Extensive gene clustering indicates that numerous serpins in close proximity on the same chromosome may have arisen as a result of duplications from a common precursor [12]; however, the evolution of these proximal genes gave way to vastly divergent functions.

Intracellular serpins of clade B are ancestral to most extracellular serpins [15,16] and each inhibitory serpin contains a highly conserved hinge region [16] within the RCL. Clade F serpins specifically share ancestry with a sea lamprey serpin. Clade P is specific to plant serpins which form a discrete clade. At the time of divergence between Viridiplantae and fungi/Metazoa groups, there was likely only one serpin gene [16]; however, the ancestral homolog from prokaryote or fungi has not yet been identified [16].

There are eight human serpin pseudogenes listed in Table 2. *SERPINA15P* has been named in succession for the A clade with the parent gene *SERPINA6* according to Ensembl and *SERPINE2* is the parent gene for *SERPINE4P*, again named in sequence of the E clade. There are ten mouse pseudogenes listed (Table 3) which remain uncharacterized.

**Table 2:** Human SERPIN genes

**Gene**	**Entrez gene ID**	**Chromosomal location**	**# Exons**	** # Amino acids**	**# Alternative transcripts**
*SERPINA1*	5265	14q32.1	5	418	10
***SERPINA2P***	**390502**	**14q32.13**	**NA**	**NA**	**NA**
*SERPINA3*	12	14q32.1	5	423	0
*SERPINA4*	5267	14q331–q32.1	5	427	0
*SERPINA5*	5104	14q32.1	6	406	0
*SERPINA6*	866	14q32.1	5	405	0
*SERPINA7*	6906	Xq22.2	5	415	0
**SERPINA7P1**	**100422644**	**Xq22.3**	**NA**	**NA**	**NA**
*SERPINA9*	327657	14q32.13	6	435	1
*SERPINA10*	51156	14q32.13	5	444	1
*SERPINA11*	256394	14q32.13	5	422	0
*SERPINA12*	145264	14q32.13	6	414	0
***SERPINA13P***	**388007**	**14q32.13**	**5**	**NA**	**NA**
***SERPINA15P***	*	**8q23.3**	**NA**	**NA**	**NA**
*SERPINB1*	1992	6p25	7	379	0
*SERPINB2*	5055	18q21.3	9	415	1
*SERPINB3*	6317	18q21.3	8	390	0
*SERPINB4*	6318	18q21.3	8	390	0
*SERPINB5*	5268	18q21.3	7	375	0
*SERPINB6*	5269	6p25	7	376	1
*SERPINB7*	8710	18q21.33	8	380	3
*SERPINB8*	5271	18q21.3	7	242	1
***SERPINB8P1***	**11029**	**6p25**	**NA**	**NA**	**NA**
*SERPINB9*	5272	6p25	7	376	0
***SERPINB9P***	**221756**	**6p25.2**	**NA**	**NA**	**NA**
*SERPINB10*	5273	18q21.3	7	397	0
*SERPINB11*	89778	**18q21.33**	8	392	0
*SERPINB12*	89777	**18q21.33**	7	405	0
*SERPINB13*	5275	18q21.3–q22	8	391	0
*SERPINC1*	462	1q23–q25.1	7	464	0
*SERPIND1*	3053	22q11.21	4	499	0
*SERPINE1*	5054	7q21.3–q22	9	402	1
*SERPINE2*	5070	2q33–q35	9	397	2
*SERPINE3*	647174	13q14.3	7	424	0
***SERPINE4P***	*	**15q12**	**NA**	**NA**	**NA**
*SERPINF1*	5176	17p13.3	8	418	0
*SERPINF2*	5345	17p13	9	491	2
*SERPING1*	710	11q12–q13.1	8	500	1
*SERPINH1*	871	11q13.5	5	418	1
***SERPINH1P1***	**158172**	**9p13.3**	**NA**	**NA**	**NA**
*SERPINI1*	5274	3q26.1	9	410	1
*SERPINI2*	5276	3q26	9	405	0
*AGT*	183	1q42.2	5	485	0

The human SERPIN family with indicated gene symbol, gene ID, chromosomal location, exon number, alternative transcript number, and number of amino acids.Gene names not italicized are used here simply to underscore that these are pseudogenes for which little *or* no information is provided. Records are from the National Center for Biotechnology Information (NCBI) gene database.

*Found in Ensembl.

**Table 3:** **Mouse *Serpin *** **genes**

***Mouse gene***	**Entrez gene ID**	**Chromosomal location**	**# Exons**	**# Amino acids**	**# Alternative transcripts**
*Serpin1a*	20700	12; 12 51.0 cM	7	413	2
*Serpin1b*	20701	12; 12 51.0 cM	5	413	1
*Serpin1c*	20702	12; 12 51.0 cM	5	413	0
*Serpin1d*	20703	12; 12 51.0 cM	5	413	0
*Serpin1e*	20704	12 E; 12	5	413	1
*Serpin1f*	68348	12 E; 12	6	411	2
Serpina2-ps*	***NA***	***12***	***NA***	***NA***	***NA***
***Serpina3d-ps***	**435318**	***12 E; 12***	***NA***	***NA***	***0***
***Serpina3e-ps***	***628883***	***12 E; 12***	***NA***	***NA***	***0***
*Serpina3a*	74069	12 E; 12	5	422	1
*Serpina3b*	271047	12 E; 12	5	420	0
*Serpina3c*	16625	12 E; 12	5	417	0
*Serpina3f*	238393	12 E; 12	6	445	2
*Serpina3g*	20715	12 E; 12	6	440	3
***Serpina3h-ps***	***546546***	***12 E; 12***	***5***	***NA***	***1***
*Serpina3i*	628900	12 E; 12	4	408	1
*Serpina3j*	238393	12 E; 12	4	420	1
*Serpina3k*	20714	12 E; 12 15.5 cM	5	418	0
***Serpina3l-ps***	***628916***	***12 E; 12***	***NA***	***NA***	***0***
*Serpina3m*	20717	12 E; 12	5	418	0
*Serpina3n*	20716	12; 12 F1	5	418	1
***Serpina4-ps***	***321018***	***12 E; 12***	***NA***	***NA***	***NA***
*Serpina5*	268591	12 F1	5	405	0
*Serpina6*	12401	12 51.0 cM	5	397	1
*Serpina7*	331535	X F1; X	6	426	2
*Agt*	11606	8 E2; 8 72.81 cM	5	482	0
*Serpina9*	71907	12	5	418	1
*Serpina10*	217847	12	5	448	1
*Serpina11*	380780	12	5	427	4
*Serpina12*	68054	12; 12 F1	5	413	0
Serpina13-ps*	***NA***	***12***	***NA***	***NA***	***NA***
*Serpinb1a*	66222	13 A4; 13 12.0 cM	8	379	0
*Serpinb1b*	282663	13 A3.3, 13 12.6 cM	7	382	0
*Serpinb1c*	380839	13 A3.3, 12 12.2 cM	7	375	1
***Serpinb1-ps***	***282665***	***13 A3.2, 13 13.76 cM***	***NA***	***NA***	***0***
*Serpinb2*	18788	1 E2.1; 1 61.1 cM	11	415	3
*Serpinb3a*	20248	1 E2.1; 1	8	387	0
*Serpinb3b*	383548	1 E2.1; 1	8	387	0
*Serpinb3c*	381286	1; 1 E1-E2	8	386	0
*Serpinb3d*	394252	1E2.1; 1	7	387	0
***Serpinb3-ps1***	***NA***	***1***	***NA***	***NA***	***NA***
***Serpinb3-ps2***	***NA***	***1***	***NA***	***NA***	***NA***
***Serpinb3-ps3***	***NA***	***1***	***NA***	***NA***	***NA***
*Serpinb5*	20724	1 E2.1; 1	8	375	2
*Serpinb6a*	20719	1313 A3.3; 13 14.0 cM	11	378	16
*Serpinb6b*	20708	13 A3.3; 13 13.78 cM	7	377	2
*Serpinb6c*	97848	13 A3.3; 13 13.99 cM	7	378	0
*Serpinb6d*	238568	13 A3.3; 13 13.94 cM	6	375	0
*Serpinb6e*	435350	13 A3.3; 13 13.98 cM	8	429	1
*Serpinb7*	116872	1; 1 D	9	380	1
*Serpinb8*	20725	1; 1 D	7	374	3
Serpinb8-ps1*	***NA***	***1***	***NA***	***NA***	***NA***
*Serpinb9*	20723	13 A3.3; 13 12.4 cM	8	374	0
*Serpinb9b*	20706	13 A3.3; 13 13.79 cM	7	377	0
*Serpinb9c*	20707	13 A3.3; 13 12.82 cM	8	387	2
*Serpinb9d*	20726	13 A3.3; 13 12.83 cM	7	377	0
*Serpinb9e*	20710	13 A3.3; 13 12.84 cM	7	377	0
*Serpinb9f*	20709	13 A3.3; 13 12.86 cM	7	377	0
*Serpinb9g*	93806	13 A3.3; 13 13.9 cM	7	377	0
*Serpinb10*	241197	1 E2. 1; 1	8	357	1
*Serpinb11*	66957	1 E2. 1; 1	8	388	0
*Serpinb12*	71869	1; 1 D	9	423	2
*Serpinb13*	241196	1 E2. 1; 1	9	389	1
*Serpinc1*	11905	1H2.1 84.6 cM	8	465	0
*Serpind1*	15160	16 A3; 16 9.5 cM	5	478	1
*Serpine1*	18787	5 G2; 5	9	402	1
*Serpine2*	20720	1 C4; 1 48.6 cM	9	397	1
*Serpine3*	319433	14 D1; 14	9	401	0
*Serpinf1*	20317	11 b5; 11	8	417	7
*Serpinf2*	18816	11 B5; 11	11	491	3
*Serping1*	12258	2 D; 2	8	504	0
*Serpinh1*	12406	7 E2; 7	6	417	1
*Serpini1*	20713	3 E3; 3	7	410	2
*Serpini2*	67931	3 E3; 3	8	405	0

The mouse serpin family with indicated gene symbol, gene ID, chromosomal location, exon number, alternative transcript number, and number of amino acids. Gene names ending in "-ps" indicate a pseudogene for which little *or* no information is provided. Records are from the National Center for Biotechnology Information (NCBI) gene database.

*=UCSC genome browser.

## Methods

Protein sequences for human serpins were accessed from Uniprot through the HUGO Gene Nomenclature Committee website (http://www.genenames.org). Sequences were retrieved from the National Center for Biotechnology Information (NCBI) gene database (http://www.ncbi.nlm.nih.gov/gene) referenced through the HUGO Gene Nomenclature Committee website (http://www.genenames.org) for humans and MGI website (http://www.informatics.jax.org) for mouse. All sequences were aligned using the most accurate settings of T-Coffee (http://tcoffee.crg.cat/) and phylogenetic trees were constructed using neighbor-joining methods with 1000 replicate bootstrap in PHYLIP 3.69 (http://evolution.genetics.washington.edu/phylip.html) (Figure 2). Expression data were determined using Genecards (http://www.genecards.org) and alternative name information was determined using HGNC (http://www.genenames.org) or MGI (http://www.informatics.jax.org).

### Human and mouse serpin isoforms

#### Clade A

Clade A serpins are classified as antitrypsin-like, extracellular proteins. They are the largest of the eight clades of extracellular serpins. The *SERPINA* clade has eleven human genes (1, 3–12) and two pseudogenes.

SERPINA1 is an inhibitory serpin formerly known as antitrypsin. It plays a role in the inhibition of neutrophil elastase [3,17].

*SERPINA2* was initially classified as a pseudogene; however, recent evidence indicates that it produces an active transcript that encodes a protein located in the endoplasmic reticulum [18]. A study that sequenced *SERPINA2* genes across multiple ethnic groups indicated that in addition to active SERPINA2 protein, there is a haplotype characterized by a partial deletion which has patterns suggestive of positive selection for loss-of-function of SERPINA2 protein. They suggest that the partial pseudogenization in humans may indicate an ongoing process of pseudogenization [19].

SERPINA3 is an inhibitory protein formerly known as antichymotrypsin. It inhibits chymotrypsin and cathepsin G [3,16]. This serpin is normally found in blood, liver, kidney, and lung.

SERPINA4 is an inhibitory protein formerly known as kallistatin (PI4), which inhibits kallikrein [20]. It is expressed in blood, liver, kidney, and heart.

SERPINA5, formerly a protein C inhibitor, inhibits active protein C. It is present in blood, kidney and liver.

SERPINA6 was formerly known as corticosteroid-binding globulin. It is a non-inhibitory protein that binds hormones, i.e., cortisol [16].

SERPINA7, formerly thyroxine-binding globulin, is involved in non-inhibitory thyroid hormone transport. It is expressed in blood, kidney, and heart.

SERPINA8 is now referred to as angiotensinogen (AGT), which is a hormone precursor. It has a distinct serpin domain (phylogenetically unrelated to other clade A members in the current analysis) and a distinct, smaller, agt domain. This particular serpin domain appears to be more closely associated with SERPINF and SERPING [21].

SERPINA9 appears to have a role in naïve B cell maintenance. Formerly called centerin, it is expressed in the plasma and liver.

SERPINA10 is an inhibitory protein responsible for inhibition of activated coagulation factors Z and XI [3]. Formerly known as protein Z-dependent proteinase inhibitor, it is expressed in blood and liver.

*SERPINA11* is likely a pseudogene and is uncharacterized.

SERPINA12, formerly vaspin, inhibits kallikrein [22] and plays a role in insulin sensitivity [23]. It appears to be expressed in plasma, platelets, liver and heart.

In the mouse (Table 3), *Serpina1* has been expanded to include six members, a**–**f. *Serpina3* has been expanded to include nine members, a**–**c and f**–**n. The other clade **a** members are orthologous to human genes. *Serpina8*, now known as *Agt* in the mouse, is vital for the development and function of the renin-angiotensin system [24]. It is orthologous to *AGT* in humans.

#### Clade B

Clade B consists of intracellular serpins, including ov-serpins, which are ancestral to the extracellular serpins [16]. Members of this subfamily have shorter C and N termini than typical A members and also lack the secretory signal peptide sequence [4]. There are 13 human genes in clade B and one pseudogene. Serpins in clade B are important in inflammation and immune system function as well as mucous production [25]. SERPINB1, B6, B7, and B9 are involved in immune system function with roles in neutrophil and megakaryocyte development [26,27], as well as in the inhibition of the cytotoxic granule protease granzyme B [28]. SERPINB3 and its close homolog B4 are inhibitors that have roles in mucous production [29] and are expressed in epithelial tissues, such as tongue, tonsils, uterus, cervix, and vagina as well as in the upper respiratory tract and thymus [30].

Despite elusive function, SERPINB3 appears to have a role in apoptotic regulation and immunity, which implicates B3 in tumor metastasis and autoimmunity [30]. SERPINB5 has been shown to inhibit metastasis as a tumor suppressor in breast and prostate cancer [30,31]. In addition, multiple serpins in the B clade have been associated with oral squamous cell carcinoma, specifically SERPINB12, SERPINB13, SERPINB4, SERPINB3, SERPINB11, SERPINB7, and SERPINB2 [32]. Less is known about SERPINB10–B13. However, recent evidence points to a role for SERPINB13 in autoimmune diabetes progression and in inflammation [33].

SERPINB1 is an inhibitor of neutrophil elastase. It was formerly called monocyte neutrophil elastase inhibitor and is expressed ubiquitously.

SERPINB2 inhibits PLAU (uPA). It was formerly called plasminogen activator inhibitor 2 (PAI2) and is expressed in blood, kidney, and liver.

SERPINB3 is a cross-class inhibitor of cathepsin L and V [34]. Formerly referred to as squamous cell carcinoma antigen 1, it is expressed in blood, immune cells, kidney, lung, heart, and brain as well as numerous mucosal cells.

SERPINB4 was formerly known as squamous cell carcinoma antigen 2; it was discovered with SERPINB3 [25]. It is a cross-class inhibitor of cathepsin G and chymase [35] and is found in plasma, platelets, kidney, and heart, as well as saliva.

SERPINB5 is a non-inhibitory protein formerly called maspin. It is likely expressed in blood, kidney, liver, lung, as well as saliva.

SERPINB6, formerly called proteinase inhibitor 6 (PI6), is an inhibitor of granule protease, cathepsin G [36]. It is expressed ubiquitously.

SERPINB7 is involved in mesangial cell proliferation [37]. Formerly called megsin, it is expressed in blood and liver.

SERPINB8 is an inhibitory protein. Formerly called proteinase inhibitor 8 (PI8), it is expressed in blood and heart.

SERPINB9 is an inhibitory protein. Formerly called proteinase inhibitor 9 (PI9), it is expressed in blood, liver, lung, and heart.

SERPINB10 is an inhibitory protein involved in hematopoietic and myeloid development [37]. Formerly called bomapin, it expressed in blood and possibly in the brain.

SERPINB11 is a non-inhibitory serpin in human but retains trypsin inhibitory activity in mice [38]. It appears not to exhibit tissue-specific expression; however, it is expressed in HEK cells.

SERPINB12 is a trypsin inhibitor formerly known as yukopin [39]. It is expressed in blood, kidney, liver, heart, and brain.

SERPINB13, formerly known as hurpin, is expressed in blood, kidney, and saliva.

In clade **b**, mouse *Serpinb1* has been expanded to include three members a**–**c; *Serpinb3* as well as *Serpinb6* have each expanded to include four members, a**–**d. In mice, *Serpinb4* is not listed; however, it appears that *SERPINB3* and *SERPINB4* are equally related to *Serpinb3a*, *Serpinb3b*, *Serpinb3c*, and *Serpinb3d*, despite the initial theory that *Serpinb3d* is the mouse homolog of human *SERPINB3* and *Serpinb3c* is the mouse homolog of *SERPINB4*. *Serpinb9* has been expanded to seven members and one pseudogene. Interestingly, *Serpinb11* is an active proteinase inhibitor, whereas the human ortholog is inactive.

#### Clade C

Serpin clade C consists of only one serpin member, SERPINC1, more commonly known as antithrombin. SERPINC1 inhibits coagulation factors IX and X [40]. It is expressed in blood, kidney, liver, lung, heart, brain, as well as saliva.

*Serpinc1* gene encodes antithrombin and is orthologous to human *SERPINC1*.

#### Clade D

Clade D has one serpin member, SERPIND1, which is an extracellular protein also known as heparin cofactor II [41]. It is an inhibitor of thrombin [42] and is expressed in blood, kidney, liver, and heart.

*Serpind1* encodes heparin cofactor II and is orthologous to *SERPIND1*.

#### Clade E

Clade E has three members, E1, E2, and E3, all of which are extracellular.

SERPINE1, also known as plasminogen activator inhibitor-1 (PAI1), inhibits thrombin. It is expressed in blood, liver, and heart.

SERPINE2 is a glial-derived nexin that is important in recovery of nerve structure and function [43]. It is expressed in blood, liver, kidney, and brain.

Little is known about the function of SERPINE3.

The mouse genes in clade **e** (*Serpine*1–3) are orthologous to human *SERPINE1–3*.

#### Clade F

There are two members in SERPIN clade F.

SERPINF1 (or pigment epithelium-derived factor (PEDF)) regulates angiogenesis and is an example of a non-inhibitory serpin. It is also thought to be a neurotrophic factor [16], and appears to be expressed in blood, liver, kidney, heart, and possibly lung.

SERPINF2, also known as α-2-antiplasmin, is an inhibitor of fibrinolysis. It is found in blood, kidney, liver, and heart.

Mouse *Serpinf1* and *f2* genes are orthologous to the human *SERPINF1* and *SERPINF2* genes, respectively.

#### Clade G

Clade G consists of one inhibitory serpin.

SERPING1 is a complement I esterase inhibitor [44] formerly called C1 inhibitor. It is expressed in blood, liver, kidney, lung, heart, and brain.

Mouse *Serping1* encodes C1 inhibitor and is orthologous to *SERPING1*.

#### Clade H

Clade H consists of one member.

SERPINH1, also known as 47-kDa heat shock protein (HSP47), does not act as a proteinase inhibitor, but rather as a chaperone for collagen [45]. It is expressed in blood, liver and heart.

Mouse *Serpinh1* encodes HSP47 and is orthologous to *SERPINH1*. Knockouts of *Serpinh1* in mice are lethal [46] and missense mutations are associated with osteogenesis imperfecta [47].

#### Clade I

Clade I consists of two extracellular proteins. Serpins in clade **I** include the following.

SERPINI1 is a neuroserpin inhibitor of PLAT (tPA), PLAU (uPA), and plasmin [48]. It is expressed in liver and possibly plasma.

SERPINI2, previously known as pancipin, has an unknown protein target but may be involved in pancreatic dysfunction [49]. It is found in platelets and plasma as well as the heart.

The genes *Serpini1* and *Serpini2* encode mouse neuroserpin and pancipin, respectively. These are orthologous to *SERPINI1* and *SERPINI2* in the human.

#### Clades J–P

Clades j**–**p represent viral, nematode, horseshoe crab, blood fluke, and plant serpins [16] and will not be described further in this update.

### Serpins associated with disease

Serpin polymorphisms have been associated with in many disease states, including blood clotting disorders, emphysema, cirrhosis, and dementia [15,16,50] as well as tumorigenesis and metastasis.

Mutations in *SERPINA1* result in a decrease in circulating α-1-antitrypsin which is associated with emphysema and hepatocellular carcinoma [51]. Serpins are implicated in regulation of the cardiovascular system. For example, SERPINA4 depletion is related to renal and cardiovascular injury [52], *SERPINA8* variations are integral to the normal function of the renin-angiotensin system and have been found to regulate blood pressure [53], and a *SERPINA10* polymorphism was found to increase the risk of venous thromboembolism [54,55]. SERPINA3 deficiency is associated with emphysema [56].

Many SERPINBs are implicated in immune function and dysfunction. In many of these cases, intracellular serpins cause autoimmune antibody production, inflammation, neutropenia, and cancer metastasis [25]. SERPINC1 deficiency has been correlated with autoimmune disease, especially in patients producing antinuclear antibodies, such as those with systemic lupus erythematosus [30]. Interestingly, a *SERPINA6* polymorphism has been associated with chronic fatigue syndrome [57], which is thought to be an immune disorder. SERPINA7 deficiency is associated with hyperthyroidism, and high SERPINA12 levels have been associated with insulin resistance [23].

Mutations in *SERPINH1*, as well as in *SERPINF1*, are associated with osteogenesis imperfecta [47,58].

Serpins appear to influence protein aggregation. In this respect, SERPINI1 expression has been correlated with dementia [4]. In addition, SERPINA5 accumulation has been identified in plaques in multiple sclerosis [59] and SERPINA3 polymerization may accelerate onset and severity of Alzheimer's disease [30].

Many serpins have been implicated in cancer progression including SERPINBs (on the 18q21 locus) in oral squamous cell carcinoma [25]. Breast and prostate cancer metastases are also closely associated with SERPINB5 [60,61]. In addition, SERPINE1 appears to have a role in tumor progression [62] and metastasis [63]. Further, SERPINI2 may play a possible role in breast and pancreatic cancer metastasis [49]. Adult gliomas have significant associations with SERPINI1 [64], although its role is unknown. In addition, SERPINI1 has also been proposed as one of five biomarkers in hepatocellular carcinoma [65]. Another potential biomarker includes SERPINA9, which has been found to be strongly expressed in B cell lymphomas [66].

### Mouse models of human disease

There are numerous mouse models used to study the role of SERPINs in disease. Some examples include knockout of *Serpinag3* used in studying T cells in immunology [67], hepatic specific knockout of *Serpinc1*, which exhibits coagulopathy [68], and *Agt* knockout to study blood pressure regulation and the renin-angiotensin system where adipocyte-specific knockout of agt caused decreased systolic blood pressure [69]. *Serpinb1* knockout mice show neutropenia [70].

### Gene variants in *SERPINS*

A large number of human variants of serpin genes have been found. For example, NCBI's dbSNP database (http://www.ncbi.nlm.nih.gov/snp) has 621 entries for SNPs of *SERPINA1* alone (accessed October 2013). In addition, several groups have developed specific databases for individual *SERPIN* genes. These include databases for *SERPINA1*[71], *SERPINC3*[72], and *SERPING1*[73]. A number of pathologies in humans have been attributed to *SERPIN* gene variants, and often multiple deleterious mutations are known for each gene. Although a full listing of disease-causing *SERPIN* mutations is beyond the scope of this review, a sample of their scope is provided here. Mutations in the *SERPINA1* gene have been linked with early-onset pulmonary emphysema, neonatal hepatitis, liver cirrhosis, and sometimes panniculitis and vasculitis [74,75]. *SERPINA5* mutations have been linked with increased papillary thyroid cancer risk [76], and mutations in *SERPINA10* have been linked to pregnancy complications [77]. Predisposition to familial venous thromboembolic disease has been linked to mutations in *SERPINC1*[78,79]. Finally, SNP variants for the *SERPING1* gene have been shown to be associated with hereditary angioedema [80].

## Conclusions

Serpins are a large class of diverse proteins, which contribute to numerous physiological and pathological conditions. Identification of serpins in immunological functions, pathology due to polymerization, and cancer metastasis underscores their diverse functions and physiological and pathological importance, and gene mutations often lead to loss-of-function and pathology in affected individuals. However, there is still much to learn about the functions and evolutionary development of serpins. Because of numerous biological functions and pathological states associated with serpins, further characterization of these proteins and mechanistic information will provide insight into potential biomarker identification and therapeutic targets.

## Competing interests

The authors declare that they have no competing interests.

## Authors’ contributions

CH carried out the sequence alignments and drafted the manuscript. BJ participated in the sequence alignment and analysis. MM reviewed mouse gene/protein data and the nomenclature for accuracy and completeness. MW reviewed human gene/protein data and nomenclature for accuracy and completeness. DT, GS and DWN reviewed and edited the manuscript. VV designed the study and reviewed data and manuscript. All authors read and approved the final manuscript.
